# When Geography Dictates Care: The Rural Gastroenterology Challenge

**DOI:** 10.14309/crj.0000000000001944

**Published:** 2025-12-22

**Authors:** Janak Bahirwani, Madhav Changela, Hareesha Rishab Bharadwaj, Dushyant Singh Dahiya

**Affiliations:** 1 Department of Gastroenterology, Kadlec Regional Medical Center, Richland, WA; 2 Elson S Floyd College of Medicine, Washington State University, Richland, WA; 3 Department of Internal Medicine, One Brooklyn Health System/Interfaith Medical Center, New York, NY; 4 Faculty of Biology Medicine and Health, The University of Manchester, Manchester, UK; 5 Division of Gastroenterology, Hepatology and Motility, The University of Kansas School of Medicine, Kansas City, KS

**Keywords:** gastroenterology, healthcare, rural, health equity, endoscopy

## INTRODUCTION

Nearly 1 in 5 Americans live in rural communities, yet access to specialty care remains disproportionately concentrated in urban centers.^1^ In gastroenterology, where timely access to procedures such as colonoscopy or endoscopic retrograde cholangiopancreatography (ERCP) can determine whether disease is caught early or progresses to complications including mortality, this imbalance represents a pressing public health inequity. Patients in rural areas often face longer travel times, delayed diagnoses, and fewer options for advanced interventions.

In the absence of gastroenterologists, general surgeons and in some regions, even primary care physicians have stepped in to provide endoscopic services. In rural Kansas, for example, surgeons perform the majority of colonoscopies, reporting confidence in routine screening but limited comfort with complex interventions.^2^ This speaks to the resilience of rural health systems, but also highlights their vulnerability when specialty expertise is needed.

Rural gastroenterology, therefore, is more than a niche concern. It reflects the broader challenges of workforce maldistribution, geographic barriers, and uneven infrastructure that define rural health care in America. At the same time, it offers a fertile ground for innovation, collaboration, and reimagined models of care-opportunities that, if embraced, could reshape not only digestive health but the very meaning of equity in our health system.

The aim of this editorial was to examine the barriers to gastroenterology care in rural America, highlight emerging solutions such as telehealth and workforce expansion, and propose strategies to bridge the gap in access and outcomes between rural and urban populations.

## BARRIERS TO GASTROENTEROLOGY HEALTH CARE ACCESS

### Geographical distance

Geography is a well-established barrier to accessing both primary and specialty care, including gastroenterology.^3^ Multiple studies demonstrate that geographic factors significantly impact the delivery of gastrointestinal (GI) health services. For patients with chronic hepatitis C virus (HCV) infection, limited access to qualified hepatologists results in fewer gastroenterology/hepatology visits and longer travel times for rural residents compared with those in urban areas.^3^ Preventive and diagnostic measures, such as hepatitis vaccination, endoscopic management of variceal bleeding, and HIV screening, remain lower in rural populations.^3^ Rural residents additionally tend to delay seeking medical attention, which contributes to greater disease severity at presentation.^4^ GI luminal cancers are distinctive in that they can be detected through endoscopic procedures. Esophageal cancer, gastric cancer, and colorectal cancers are typically diagnosed during endoscopic evaluation. Evidence from a retrospective cohort study using the Florida Cancer Data System, a population-based cancer incidence registry, demonstrated that greater distance from care and residence in socioeconomically deprived areas correlate with more advanced stages of cancer at diagnosis.^5^ In addition, patients with high accessibility to the hospitals performing procedures like ERCP are more likely to undergo the treatment.^6^

### Workforce maldistribution

Challenges in ensuring access to health care in rural areas are well documented and include healthcare provider shortages, as well as hospital and nursing home closures.^7^ Studies report a disproportionate distribution of specialists-including gastroenterologists and surgeons-with higher concentrations in urban areas and lower availability in rural communities.^8,9^ Although nearly 20% of the U.S. population resides in rural areas, only about one-tenth of physicians practice there.^4^ Federal agencies and modeling analyses project a continued shortage of both primary care providers and specialists, including gastroenterologists, in rural regions in the coming years.^10–12^ Contributing factors include rising demand from an aging population, the growing burden of metabolic dysfunction–associated steatotic liver disease, increasing prevalence of alcohol-associated liver disease, and an aging physician workforce.^11^ Most training programs are concentrated in urban and suburban centers, and even trainees with interest in rural practice may feel inadequately prepared to address the unique challenges of rural healthcare delivery.^10^In addition, for middle and high-school students in rural communities who express an interest in science, the scarcity of physician role models limits early exposure to medicine as a potential career path.^10^

### Limited specialized services

Significant disparities have been documented in access to procedural interventions such as esophagogastroduodenoscopy, colonoscopy, ERCP, and abdominal surgery between rural and urban populations.^9,13–15^ Rural patients, including pediatric population, have consistently been shown to have reduced access to specialized endoscopic and surgical services.^15,16^ Similar inequities extend to liver transplantation, the cornerstone therapy for end-stage liver disease. Studies indicate that rural residents with chronic liver disease are less likely to receive liver transplantation compared with their urban counterparts. Contributing factors include the concentration of liver transplant centers in urban areas, limited access to reliable transportation, and delays in referral.^17,18^ Preventive care also shows geographic gaps. A population-based cross-sectional study found that individuals residing in rural areas had significantly lower odds of undergoing screening colonoscopy compared with urban residents (odds ratio, 0.81; 95% confidence interval, 0.66–0.99; *P* = 0.047).^19^ These findings underscore the persistent challenges rural communities face in accessing both specialized interventions and preventive services.

## POTENTIAL SOLUTIONS TO GASTROENTEROLOGY HEALTHCARE ACCESS

### Telehealth

Telehealth has become a transformative tool in gastroenterology, offering innovative approaches to improve access, continuity of care, and patient outcomes across a wide range of digestive diseases, especially after the COVID-19 pandemic.^20^ It addresses longstanding barriers such as limited transportation and delays in referral.^21^ Studies demonstrate that telehealth reduces time from referral to evaluation and waitlisting, and is increasingly being integrated with mobile health applications to enhance communication between patients and providers. Although adoption of telehealth has become nearly universal, use still varies by region and practice type. Digital health interventions have been associated with improvements in quality of care and quality of life. A randomized controlled trial at Mount Sinai Medical Center evaluated the HealthPROMISE application among 320 patients with inflammatory bowel disease and demonstrated marked improvements in quality of care (28% vs 9%, *P* < 0.01) and quality of life, as well as a significant reduction in emergency department visits and hospitalizations after 1 year of use (25% vs 3%, *P* = 0.03).^22^ Similar mobile applications are being developed for conditions such as celiac disease to support monitoring and self-management.^23^ A program launched under the leadership of Dr Corey Siegel provides multidisciplinary, telehealth services for patients with inflammatory bowel diseases and a 2-page summary letter is sent to the referring provider to help guide management.^24^

Telemedicine has also been particularly impactful in expanding access to HCV care, especially in rural areas and among high-risk populations such as people who use drugs and incarcerated individuals. The Extension for Community Healthcare Outcomes model represents one of the most successful approaches, creating partnerships between academic centers, public health systems, correctional facilities, and rural clinics to deliver protocol-driven care and provider education.^25^ This model has improved HCV screening, linkage to care, and treatment outcomes in underserved areas.^26^ A study conducted at an Italian addiction center further highlighted its effectiveness, showing that telemedicine-supported HCV care achieved a sustained virologic response rate of 98.5%, with 100% linkage to care among people who use drugs.^27^ Integrating addiction medicine and hepatology through telemedicine-based multidisciplinary models holds additional promise for improving adherence to guideline-directed therapy and optimizing outcomes in populations with overlapping substance use disorder and liver disease.^28^

Beyond direct patient care, telemedicine also serves as a valuable platform for medical education. Virtual case discussions, remote supervision, and access to expert-led programs can enhance resident training, particularly in institutions serving rural and underserved communities.^29^ As gastroenterology moves toward value-based care, telemedicine and digital health innovations offer powerful tools to track outcomes, improve access, and deliver specialized, patient-centered care to populations that have historically been difficult to reach. The likelihood of continued telehealth reimbursement for gastroenterology services in rural areas in the United States depends on both federal and state legislative actions, with no permanent nationwide guarantee as of September 2025.

### Workforce expansion and training

Medical schools play a central role in shaping the future rural workforce, with direct influence over student recruitment, admissions policies, rural-oriented curricula, clinical learning experiences, faculty values, and advanced procedural training. Evidence suggests that these factors significantly affect the likelihood of graduates entering rural practice. Physicians raised in rural communities are 2.3 times more likely than their urban-raised peers to choose rural practice, and 2.5 times more likely to remain in rural practice over time.^30^ Similarly, residents who participate in rural family medicine rotations demonstrate a higher likelihood of pursuing rural careers.^29,30^ Encouraging mentorship through creation of programs, especially for underrepresented students in medicine, is essential to cultivate a diverse and inclusive cadre of leadership.^31^

Financial considerations also influence workforce distribution. With rising levels of educational debt, many physicians rely on programs such as Public Service Loan Forgiveness and the National Health Service Corps. While Public Service Loan Forgiveness supports family physicians planning careers in public service, National Health Service Corp participation is more strongly associated with eventual practice in underserved communities.^32^ Loan repayment programs targeted at rural states, such as those in Colorado, often attract physicians who may already have intended to work in rural areas, but these programs can influence the specific community chosen and play an important role in provider retention.^33^ However, long-term retention is often more affected by family considerations and professional satisfaction than by financial incentives alone.

Expanding recruitment efforts to include trainees from underserved or marginalized backgrounds may further strengthen the pipeline of providers interested in working outside major academic and transplant centers.^18^ In addition, restructuring training pathways may help address workforce shortages in subspecialties. For example, decoupling advanced hepatology from gastroenterology and allowing physicians to enter hepatology training directly after internal medicine residency could shorten the training period and potentially draw more physicians into the field. International Medical Graduates (IMGs) are approximately one quarter of the physician workforce and streamlining processes for IMGs, especially policies governing visas and immigration as well as expansion of the J-1 waiver program will also help in recruitment of IMGs to rural communities and lead to improvement in barriers to GI care.^34^

### Transportation

Providing reliable transportation for rural patients is a critical component of improving health care quality. One potential strategy is the incorporation of neighborhood deprivation indices into liver transplant evaluations, which would allow transplant centers to identify patients who may benefit from additional support services such as social workers, case managers, and patient navigators.^18^ These personnel can play a pivotal role in addressing specific barriers-financial, educational, cultural, and social-that disproportionately affect patients from more deprived communities. Evidence also highlights the importance of geographic accessibility in procedural care. In our population-based study, patients who lived within a 30- to 45-minute drive of an ERCP-performing hospital were significantly more likely to receive ERCP as the preferred initial biliary drainage procedure.^6^ More broadly, distance to care remains a persistent barrier to colorectal cancer screening, contributing to disparities in early detection and outcomes among rural populations.^35^ To reduce these disparities, future interventions must not only expand local screening capacity but also provide targeted support to help patients and providers navigate geographic barriers within their community contexts.

### Infrastructure and endoscopic services

GI endoscopy units, both freestanding and affiliated with ambulatory surgical centers, are increasing in number, proved to be instrumental in addressing GI issues in rural population. In the late 20th century, the “round-trip transfer” model was tested for therapeutic ERCP, showing that patients could safely undergo procedures at specialized centers and return the same day to rural hospitals if carefully selected.^36^ A similar approach in Minnesota used ambulance transfers for same-day endoscopy with good outcomes.^37^ In India, the Rural Health Care Project deployed a mobile hospital bus equipped with an endoscopy unit and supported by a telemedicine van. Over 30,000 procedures have been performed across nearly 5,000 villages, serving over 10 million people, with high satisfaction reported.^38^ When developing ambulatory endoscopy centers, key considerations include local demographics, disease prevalence, access to tertiary care for emergencies, and patient affordability.^39^

## LOOKING AHEAD TOWARD THE FUTURE

The rural landscape, though resource-constrained, is also a fertile ground for innovation. Expanding access to GI care will require more than increasing procedure capacity-it calls for reimagining how care is delivered and how clinicians are supported. Policy advocacy must prioritize sustained telehealth reimbursement, expansion of loan repayment and J-1 waiver programs, and investment in rural endoscopy infrastructure. At the same time, a deliberate workforce strategy that recruits trainees with rural backgrounds and modernizes training pathways is essential to closing long-standing gaps.

Technology will continue to shape the future. Telehealth, mobile endoscopy, and digital health applications are already narrowing geographic divides, while artificial intelligence and remote monitoring promise to further optimize access and outcomes. Strengthening partnerships between academic centers and rural hospitals can expand training, research, and shared expertise, ensuring innovation extends beyond urban hubs.

Equally important is building a culture that sustains the clinicians who provide this care. Addressing physician burnout by reducing administrative burdens, improving efficiency of electronic health records, and fostering supportive workplace environments will be vital to retaining physicians in rural practice. By committing to equity, innovation, and physician well-being, gastroenterology can lead the way in ensuring geography no longer dictates digestive health outcomes.

Rural gastroenterology is both a challenge and an opportunity. Addressing disparities in access demands collaboration across health systems, policymakers, and the GI community. By reimagining care delivery and fostering a workforce committed to underserved regions, gastroenterology can lead the way in reducing healthcare inequities and ensuring that geography does not determine health outcomes.

## DISCLOSURES

Author contributions: J. Bahirwani formulated the idea, literature review and drafting of manuscript. M. Changela literature review and drafting of manuscript. H.R. Bharadwaj literature review and manuscript editing. D.S. Dahiya formulated the idea and manuscript editing and is the article guarantor.

Financial disclosure: None to report.

## ABBREVIATIONS:

CRC: colorectal cancer
EC: esophageal cancer
ECHO: extension for community healthcare outcomes
EGD: esophagogastroduodenoscopy
ERCP: endoscopic retrograde cholangiopancreatography
GC: gastric cancer
GI: gastrointestinal/gastroenterology
HCV: hepatitis C virus
LT: liver transplant
MASLD: metabolic dysfunction–associated steatotic liver disease
NHSC: National Health Service Corps
PSLF: public service loan forgiveness
QOC: quality of care
QOL: quality of life
